# Menstrual cycle changes: A cross-sectional study of Saudi females following SARS-CoV-2 infection

**DOI:** 10.1371/journal.pone.0279408

**Published:** 2022-12-20

**Authors:** Youssef A. S. Abdel-Moneim, Hussam Y. Alghamdi, Abdulaziz M. Alrashed, Amjad M. Jawhari, Suhaib M. M. Bukhari, Nirmeen M. M. Bukhari, Ahmed S. Abdel-Moneim

**Affiliations:** 1 Faculty of Medicine, Beni-Suef University, Beni-Suef, Egypt; 2 College of Medicine, Taif University, Al-Taif, Saudi Arabia; 3 College of Medicine, Jeddah University, Jeddah, Saudi Arabia; 4 College of Medicine, King Abdulaziz University, Jeddah, Saudi Arabia; University of Hail, SAUDI ARABIA

## Abstract

Temporary changes in the menstrual cycle have recently been reported following SARS-CoV-2 vaccination. In the current study, we aimed to screen menstrual cycle changes following SARS-CoV-2 infection in Saudi Arabia. The type and duration of these changes have been screened in relation to the severity of coronavirus disease symptoms and vaccination status. In total, 956 individuals responded: sixty-nine did not get the COVID-19 vaccine, while the remaining were vaccinated with either a single dose of ChAdOx1 vaccine (n:45) or BNT162b2 vaccine (n: 142) or two doses of the vaccine (n:700) using BNT162b2 (n:477), ChAdOx1 (n:89) or ChAdOx1/ BNT162b2 (n:134). Approximately 26.1% (18/69) of the subjects who did not receive the SARS-CoV-2 vaccine and 15.3% (29/188) and 26.4% (185/700) of the subjects who received single and double doses of the vaccines, respectively, reported menstrual cycle changes. The persistence of menstrual cycle changes for more than six months was reported by 6.4% (61/956) of the participants. These changes were significantly correlated with the severity of COVID-19 infection. We concluded that menstrual cycle changes, associated with COVID-19 infection, increase due to the severity of COVID-19 infection. Thus, menstrual cycle changes are among the long-term effects associated with COVID-19 infection.

## Introduction

SARS-CoV-2 is the causative agent of coronavirus disease (COVID-19), which constitutes the first coronavirus pandemic worldwide. Most patients with infection passed the disease silently or in an asymptomatic form, which was the case with most of the infected patients. However, severe and critical forms of the disease were reported in 15% and 5% of infected patients, respectively. From its first emergence on 19 December 2019 until 29 November 2022, there have been more than 639 million laboratory-confirmed SARS-CoV-2 infections, including 6,615,258 deaths [1].

Globally, most countries have been put on lockdown due to the current pandemic. It resulted in a global stressor and panic for the public as the virus spread rapidly and led to hospitalizations and deaths. SARS-CoV-2 vaccines are currently available, and 13,042,112,489 vaccine doses have been administered so far [1]. In Saudi Arabia, BNT162b2, ChAdOx1, and Moderna vaccines were approved for use as SARS-CoV-2 vaccination. Although the vaccines are safe and efficacious in preventing the severe form of the disease and mortalities, like other vaccines, they induce post-vaccinal side effects, including some reports about menstrual cycle changes [2–6].

The normal menstrual cycle indicates that females are not subjected to any stress and that their ovaries and pituitary glands are functioning appropriately. In contrast, menstrual cycle irregularities include menorrhagia, dysmenorrhea, or shortened or prolonged menstrual periods [7]. Such menstrual changes are linked to the level of stress encountered by female populations, as historically reported [8–10]. Menstrual aberrations can result from either psychological or physical causes and can last for a short or extended period [10,11]. Gonadotropin-releasing hormone (GnRH), luteinizing hormone (LH) and estradiol (E2) are associated with stress-related changes in the menstrual cycle, which range from delayed ovulation to amenorrhea [12]. COVID-19 leads to an increased level of anxiety about getting ill. In addition, few studies have investigated the influence of COVID-19 infection and vaccination on menstrual changes [2,12–14].

So far, inconsistent findings have been reported regarding alterations in menstrual cycle characteristics following COVID-19 infection or vaccination [12,15–20]. Moreover, most of these studies have not considered the effect of COVID-19 infection, disease severity in affected individuals, the effect of the type and number of doses of the COVID-19 vaccine in infected individuals, and time span of the menstrual cycle changes. In addition, limited information is available on the Saudi population regarding the effect of COVID-19 infection on the menstrual cycle. Accordingly, the current study aims to screen females who have been infected with SARS-CoV-2, regardless of their vaccinal status, to address their complaints of menstrual cycle changes and how long the changes persisted after infection. The possible effects of age, the severity of COVID-19 infection, vaccination status, and time of vaccination to the time of infection were among the objectives of the current study.

## Methods

### Ethics statement

The study has been reviewed and approved by the Taif University Ethical Committee, with approval No. 43-002/2021. Completing the questionnaire has been considered as an informed written consent for participating in the study.

### Subjects

The inclusion criteria for filling out the questionnaire were females infected with SARS-CoV-2 and aged between 18 to 40 years. The participants were asked to fill out their demographic data and the degree of severity of their COVID-19 infection, which was graded into mild (mild disease manifestations, 1–3 days), moderate (tolerable disease manifestations, 4–7 days), severe (severe signs but hospitalization not required, 8–14 days or more) and critical (severe signs and hospitalization required). The questionnaire included questions about SARS-CoV-2 vaccination status, the number of vaccine doses, the type of vaccine, and exposure to SARS-CoV-2 infection to the time of getting the vaccine (before the first dose, after the first dose, and after the second dose). The participants have been then asked about the presence or absence of menstrual cycle changes. If they answered yes, then they have asked about the symptoms (menorrhagia, dysmenorrhea, or increased days of the cycle) they encountered and how long these symptoms lasted (>2 months to >6 months) (S1 File). The questionnaire was a modified version from recently published ones [15,21]. Different social media platforms (Twitter, Snapchat, and WhatsApp) have been used to distribute the online questionnaire to Saudi nationals and residents.

### Statistical analysis

The respondents’ number and percentage in each group have been estimated. The effects of age, vaccine status, time of infection to time of vaccination, and severity of the disease (mild, moderate, severe, or serious case) on menstrual cycle changes and their duration, have been analyzed using crosstab. Chi-square and Spearman rho correlations using the SPSS software program have been used to screen the differences and correlations among these variables.

## Results

The total number of participants was 956, aged from 18 to 40 years old. Most respondents did not suffer from any menstrual cycle change (724/956, 75.7%), and the remaining (232/956, 24.3%) reported changes, which lasted from two to more than six months. Among the respondents with complaints of menstrual cycle changes, there was neither a significant difference nor a meaningful correlation between the respondent’s age and duration of changes. Meanwhile, there was a highly significant variation among different age groups (P < 0.003) (Table 1). Delayed menstrual cycle was the highest in females aged 18–20 years (42/214, 19.6%), and excessive bleeding was higher in age groups 21–30 (19/520, 3.7%) and 31–40 (7/222, 3.2%) compared to the 18 to 20-year-old group (2/214, 0.9%). Extended menstrual cycle was the highest in the 31 to 40-year-old group (11/222, 5%) (Table 1).

**Table 1:** Effect of age on the development of menstrual cycle changes following SARS-CoV-2 infection.

Age (Years)		None ^a^	Description of menstrual changes ^b^ n:232				Duration of menstrual cycle changes after COVID-19 infection^c^ (Months), n:232						Total
			Menorrhagia	Dysmenorrhea	More days than usual	Delayed menstrual cycle than normal	2	3	4	5	6	>6	
18–20	No	2	8	6	42	5	17	17	7	5	3	9	214
	%	0.9	3.7	2.8	19.6	2.3	7.9	7.9	3.3	2.3	1.4	4.2	
21–30	No	19	31	7	70	9	38	20	21	9	1	38	520
	%	3.7	6.0	1.3	13.5	1.7	7.3	3.8	4.0	1.7	.2	7.3	
31–40	No	7	6	11	23	2	17	9	3	2	2	14	222
	%	3.2	2.7	5.0	10.4	.9	7.7	4.1	1.4	.9	.9	6.3	
Total	No	28	45	24	135	16	72	46	31	16	6	61	956
	%	2.9	4.7	2.5	14.1	1.7	7.5	4.8	3.2	1.7	.6	6.4	

^a^ None: No menstrual cycle changes were detected.

^b^ Chi-square results showed highly significant variation in the type of menstrual cycle changes among different age groups (P < 0.003). No significant correlation has been obtained based on the Spearman rho correlation.

^C^ The duration of menstrual cycle changes: Non-significant variation and no correlation.

In the different vaccinated groups, neither a significant difference nor a significant correlation was observed between the type of vaccine and both menstrual changes and their duration (Table 2). In addition, the relation between the time of infection and the time of vaccination showed a similar non-significant profile (Table 3).

**Table 2:** Effect of the time of infection on the time of vaccination on the development of menstrual cycle changes following infection with SARS-CoV-2.

Vaccination Status		None ^a^	Description of menstrual changes ^c^, n:232				Duration of menstrual cycle changes after COVID-19 infection ^b^ (Months), n:232						Total
			Menorrhagia	Dysmenorrhea	More days than usual	Delayed menstrual cycle than normal	2	3	4	5	6	>6	
Not vaccinated	No	51	2	5	2	9	7	3	2	1	0	5	69
	%	73.9	2.9	7.2	2.9	13.0	10.1	4.3	2.9	1.4	0.0	7.2	
A single dose of BNT162b2	No	120	7	6	1	8	6	6	3	2	0	5	142
	%	84.5	4.9	4.2	0.7	5.6	4.2	4.2	2.1	1.4	0.0	3.5	
A single dose of ChAdOx1	No	39	0	2	0	4	4	0	0	0	1	1	45
	%	86.7	.0	4.4	0.0	8.9	8.9	0.0	0.0	0.0	2.2	2.2	
Two doses of BNT162b2	No	347	13	22	15	80	37	23	18	10	5	37	477
	%	72.7	2.7	4.6	3.1	16.8	7.8	4.8	3.8	2.1	1.0	7.8	
Two doses of ChAdOx1	No	67	4	3	1	14	7	4	3	1	0	7	89
	%	75.3	4.5	3.4	1.1	15.7	7.9	4.5	3.4	1.1	0.0	7.9	
ChAdOx1 BNT162b2	No	100	2	7	5	20	11	10	5	2	0	6	134
	%	74.6	1.5	5.2	3.7	14.9	8.2	7.5	3.7	1.5	0.0	4.5	
Cumulative	No	724	28	45	24	135	72	46	31	16	6	61	956
	%	75.7	2.9	4.7	2.5	14.1	7.5	4.8	3.2	1.7	0.6	6.4	

^a^ None: No menstrual cycle changes.

^b, c^ Chi-square and the Spearman rho correlations did not show any significant variations.

**Table 3:** Effect of time of infection on the time of vaccination on the development of menstrual cycle changes following SARS-CoV-2 infection.

Time of infection in relation to vaccination		None ^a^	Description of menstrual changes ^b^, n:232				Duration of menstrual cycle changes after COVID-19 infection ^c^ (Months), n:232						Total
			Menorrhagia	Dysmenorrhea	More days than usual	Delayed menstrual cycle than normal	2	3	4	5	6	>6	
Infected and not vaccinated	No	51	2	5	2	9	7	3	2	1	0	5	69
	%	73.9	2.9	7.2	2.9	13.0	10.1	4.3	2.9	1.4	.0	7.2	
Infection before the first dose of the vaccine	No	433	15	21	13	84	38	27	18	7	4	39	566
	%	76.5	2.7	3.7	2.3	14.8	6.7	4.8	3.2	1.2	.7	6.9	
Infection after the first dose of the vaccine	No	192	9	14	7	33	22	13	8	5	2	13	255
	%	75.3	3.5	5.5	2.7	12.9	8.6	5.1	3.1	2.0	.8	5.1	
Infection after the second dose of the vaccine	No	48	2	5	2	9	5	3	3	3	0	4	66
	%	72.7	3.0	7.6	3.0	13.6	7.6	4.5	4.5	4.5	.0	6.1	
Cumulative	No	724	28	45	24	135	72	46	31	16	6	61	956
	%	75.7	2.9	4.7	2.5	14.1	7.5	4.8	3.2	1.7	.6	6.4	

^a^ None: No menstrual cycle changes.

^b, c^ Chi-square and the Spearman rho correlations did not show any significant variations.

Significant variation (P < 0.008) was reported among participants who suffered from different COVID-19 disease severities (Table 4). A significant reverse correlation was detected between COVID-19 disease severity and the type of menstrual cycle changes (P < 0.001, R = -0.134). Similarly, a statistically significant correlation (P < 0.001, R = -0.144) has been found between disease severity and the duration of menstrual cycle changes. The milder the disease severity, the lower the frequency of patients who reported menstrual cycle changes. Delayed menstrual cycle (135/956, 14.1%) was among the most frequently detected changes, followed by dysmenorrhea (45/956, 4.7%), excessive bleeding (28/956, 2.9%) and extended cycle (24/956, 2.5%) (Table 4).

**Table 4:** Effect of COVID-19 severity on the development of menstrual cycle changes following SARS-CoV-2 infection.

Disease severity		None^a^	Description of menstrual changes ^b^, n:232				Duration of menstrual cycle changes after COVID-19 infection ^c^ (Months), n:232						Total
			Menorrhagia	Dysmenorrhea	More days than usual	Delayed menstrual cycle than normal	2	3	4	5	6	>6	
Asymptomatic	No	93	0	1	1	10	1	3	4	1	0	3	105
	%	88.6	0.0	1.0	1.0	9.5	1.0	2.9	3.8	1.0	.0	2.9	
Mild	No	196	6	10	3	33	19	10	7	4	3	9	248
	%	79.0	2.4	4.0	1.2	13.3	7.7	4.0	2.8	1.6	1.2	3.6	
Moderate	No	282	10	21	13	50	28	20	14	7	2	23	376
	%	75.0	2.7	5.6	3.5	13.3	7.4	5.3	3.7	1.9	.5	6.1	
Severe but did not require hospitalization	No	144	10	11	6	36	22	11	6	4	1	19	207
	%	69.6	4.8	5.3	2.9	17.4	10.6	5.3	2.9	1.9	.5	9.2	
Critical and required hospitalization	No	9	2	2	1	6	2	2	0	0	0	7	20
	%	45.0	10.0	10.0	5.0	30.0	10.0	10.0	0.0	0.0	0.0	35.0	
Cumulative	No	724	28	45	24	135	72	46	31	16	6	61	956
	%	75.7	2.9	4.7	2.5	14.1	7.5	4.8	3.2	1.7	.6	6.4	

^a^ None: No menstrual cycle changes.

^b^ Chi-square, P < 0.008, the Spearman rho correlation, P < 0.001. R = -0.134.

^c^ Chi-square, P < 0.001, the Spearman rho correlation, P < 0.001, R = -0.144.

## Discussion

Approximately one-fourth of the females, who have participated in the current study, suffered from menstrual cycle changes. One of the strengths of this study lies in its estimation of the approximate duration of menstrual changes. The survey was anonymous to reduce the social reluctance of people who would not like to report socially embarrassing experiences as considered in Saudi culture.

The reported menstrual cycle changes following SARS-CoV-2 infection continued from two months to more than six months. Among the participants, 6.4% showed persistent complaints for more than 6-months. Although many studies have shown that COVID-19 vaccination is associated with menstrual cycle changes [2,22,23], the current study did not find any significant variation when using different vaccines with single or double doses. Our findings support previous reports which have indicated no association between COVID-19 vaccination and the development of menstrual cycle changes [20,24]. Most studies reporting menstrual cycle changes following the vaccination have demonstrated a short-term effect [23,25].

Some recent studies have also reported short-term menstrual cycle changes following COVID-19 infection [15]. These effects can be attributed to fear or stress from the vaccination [7,11,18,26] rather than an actual effect on the female reproductive tract. Accordingly, we assume that the long-term menstrual cycle changes are due to COVID-19 infection rather than vaccination. The menstrual cycle changes detected in the current study were positively correlated with COVID-19 severity, which is in line with a recent finding [27].

Furthermore, psychological distress affects women’s mood and correlates with menstrual cycle changes. Stress shows inhibitory effects on the production of GnRH, LH, estrogen, and progesterone by the ovary [26,28,29]. Compared to men, women are more susceptible to negative psychological responses, including stress, anxiety, and depression [30,31]. During the SARS-CoV-2 pandemic, an alteration of human behavior based on the fear and panic of being infected was reported in previous studies [21,31,32]. Such a mechanism explains the short-term effect on the menstrual cycle; however, in the current study, the observed symptoms of menstrual cycle changes extended for more than six months from the time of infection in 6.4% of the respondents.

This long duration of menstrual changes is a possible attributable effect of the SARS-CoV-2 infection SARS-CoV-2 on the ovaries or reproductive tract. This finding agrees with the recent findings that revealed that COVID-19 affects the reproductive system [33,34]. It has been speculated that SARS-CoV-2 infects the ovaries and endometrium, with subsequent alterations in their functions [33]. SARS-CoV-2 uses ACE-2 cell receptors to enter the target cells. ACE-2 is present in the respiratory tract and different tissues, including tissues of the female reproductive system (ovary, uterus, and vagina) [35–37]. Alteration of the expression of such receptors has been reported during different phases of the menstrual cycle [38]. ACE2 expression in cells is abundant in the secretory phase, while interference occurs with the Ang-II local homeostasis [39]. The ACE2 receptor expression in ovaries engages in follicular development [40,41], augments the finding that SARS-CoV-2 can directly impact female reproductive functions [34]. Thus, SARS-CoV-2 infection might be responsible for extending menstrual changes beyond six months, as detected in the current study.

## Limitations

The main limitation of the current study is self-reported data, which could be subjected to bias and incur measurement errors in the description of menstrual cycle changes by the respondents. The second main limitation is sampling bias.

## Supporting information

S1 File Supplementary 1: Questionnaire used in the study. (DOCX)
