# Correction: Menstrual cycle changes: A cross-sectional study of Saudi females following SARS-CoV-2 infection

**DOI:** 10.1371/journal.pone.0301574

**Published:** 2024-03-27

**Authors:** Youssef A. S. Abdel-Moneim, Hussam Y. Alghamdi, Abdulaziz M. Alrashed, Amjad M. Jawhari, Suhaib M. M. Bukhari, Nirmeen M. M. Bukhari, Ahmed S. Abdel-Moneim

The affiliation for the seventh author is incorrect. Ahmed S. Abdel-Moneim is not affiliated with #1 but with #2: College of Medicine, Taif University, Al-Taif, Saudi Arabia.

## References

[1] Abdel-Moneim YAS, Alghamdi HY, Alrashed AM, Jawhari AM, Bukhari SMM, Bukhari NMM, et al. (2022) Menstrual cycle changes: A cross-sectional study of Saudi females following SARS-CoV-2 infection. PLoS ONE 17(12): e0279408. doi: 10.1371/journal.pone.0279408 36538566 PMC9767340

