# Supplementary material for: Menstrual cycle changes: A cross-sectional study of Saudi females following SARS-CoV-2 infection
Source: PLoS One. 2022 Dec 20;17(12):e0279408. doi: 10.1371/journal.pone.0279408 (PMC9767340; doi:10.1371/journal.pone.0279408)
Supplement: S1 File — (DOCX) [file pone.0279408.s001.docx]

**Questionnaire**

In the current study, we aim to determine the menstrual cycle changes that happen after infection with COVID-19.

Females aged 18–40 years who have suffered from laboratory-confirmed COVID-19 infection are the only eligible subjects to fill out this questionnaire. This research bears the approval of the Scientific Research Ethics Committee at Taif University No. 43-002 and date 08/17/2021. Filling out the questionnaire constitutes an informed written consent to participate in the study.

**Do you agree to participate in the study and confirm that you are among the eligible subjects [females aged 18–40 years previously infected with COVID-19]?**

- YES
- NO

**Kindly confirm that you have experienced a laboratory-confirmed COVID-19 infection during the last year.**

- Yes
- No

**Age**

- 18–20 years
- 21–30 years
- 31–40 years

**Which type of vaccine have you taken?**

- Did not receive any vaccine
- Single dose of BNT162b2 [single dose of Pfizer vaccine]
- Single dose of ChAdOx1 [single dose of AstraZeneca vaccine]
- Two doses of BNT162b2 [two doses of Pfizer vaccine]
- Two doses of ChAdOx1 [two doses of AstraZeneca vaccine]
- ChAdOx1 BNT162b2 [AstraZeneca and Pfizer vaccines]

**Could you kindly let us know which of the following describes your COVID infection and COVID vaccination?**

- Infected and not vaccinated
- Infection before the first dose of the vaccine
- Infection after the first dose of the vaccine
- Infection after the second dose of the vaccine

**Kindly describe the severity of your COVID infection.**

- Asymptomatic [no signs]
- Mild [mild clinical symptoms without changes in X-ray or CT and normal respiratory rate]
- Moderate [moderate respiratory symptoms associated with changes observed in CT scans and normal respiratory rate]
- Severe but did not require hospitalisation [Severe respiratory distress, with a respiratory rate ≥30/min and resting blood oxygen saturation ≤ 93]
- Critical and required hospitalisation [including mechanical ventilation]

**Did you suffer from menstrual cycle changes after a COVID infection?**

**YES**

**NO**

If yes (suffered from any menstrual cycle changes listed in the previous question), kindly answer the following questions:

**What are the types of menstrual changes?**

- Menorrhagia
- Dysmenorrhea
- Extended cycle
- Delayed menstrual cycle

**How long did the changes in menstrual cycles continue?**

- 2 months
- 3 months
- 4 months
- 5 months
- 6 months
- More than 6 months
